# Solving the molecular distance geometry problem with inaccurate distance data

**DOI:** 10.1186/1471-2105-14-S9-S7

**Published:** 2013-06-28

**Authors:** Michael Souza, Carlile Lavor, Albert Muritiba, Nelson Maculan

**Affiliations:** 1 Department of Statistics and Applied Mathematics, Federal University of Ceará, Ceará, 60455-760, Brazil; 2 Institute of Mathematics, Statistics and Scientific Computing, University of Campinas, Campinas, 13083-859, Brazil; 3 Institute Alberto Luiz Coimbra, Federal University of Rio de Janeiro, Rio de Janeiro, 21941-972, Brazil

## Abstract

We present a new iterative algorithm for the molecular distance geometry problem with inaccurate and sparse data, which is based on the solution of linear systems, maximum cliques, and a minimization of nonlinear least-squares function. Computational results with real protein structures are presented in order to validate our approach.

## Background

The knowledge of the protein structure is very important to understand its function and to analyze possible interactions with other proteins. Different methods can be applied to acquire protein structural information. Until 1984, the X-ray crystallography was the ultimate tool for obtaining information about protein structures, but the introduction of nuclear magnetic resonance (NMR) as a technique to obtain protein structures made it possible to obtain data with high precision in an aqueous environment much closer to the natural surroundings of living organism than the crystals used in crystallography [1].

The NMR technique provides a set of inter-atomic distances for certain pairs of atoms of a given protein. The molecular distance geometry problem (MDGP) arises in NMR analysis context. The MDGP consists of finding one set of atomic coordinates such that a given list of geometric constraints are satisfied [2]. Formally, the molecular distance geometry problem can be defined as the problem of finding Cartesian coordinates $x_{\text{1}},\;.\;.\;.\;,x_{n}\in {\mathbb{R}}^{\text{3}}$ of atoms of a molecule such that *l_ij _*≤ ||*x_i _*- *x_j_*|| ≤ *u_ij_*, ∀(*i*, *j*) ∈ *E*, where the bounds *l_ij _*and *u_ij _*for the Euclidean distances of pairs of atoms (*i*, *j*) ∈ *E *are given *a priori *[3].

As suggested by Crippen and Havel [3], the MDGP can also be formulated as the global optimization problem of minimizing the function

$$f\left(x\right)=\underset{\left(i,j\right)\;\in \;E}{\sum }p_{ij}\left(x_{i}-x_{j}\right),$$

where the pairwise function $p_{ij}:{\mathbb{R}}^{n}\to \mathbb{R}$ is defined by

$$p_{ij}\left(x\right)=\text{max}\left\{\frac{l_{ij}^{2}-{||x||}^{2}}{l_{ij}^{2}},\frac{{||x||}^{2}-u_{ij}^{2}}{u_{ij}^{2}},0\right\}.$$

Clearly, $x=\left(x_{\text{1}},\;.\;.\;.\;,x_{n}\right)\in {\mathbb{R}}^{\text{3}n}$ solves the MDGP if, and only if, *x *is a global minimizer of *f *and *f*(*x*) = 0.

An overview on methods applied to the MDGP is given in [4] and a very recent survey on distance geometry is given in [5].

Particular cases of the MDGP can be solved in a relatively easy way. For instance, when we know all distances *d_ij _*= ||*x_i _*- *x_j_*||, i.e., *d_ij _*= *l_ij _*= *u_ij _*and *E *= {1, 2, ..., *n*}^2^, a solution can be obtained by factoring the distance matrix *D *= [*d_ij_*]. Assuming that *D *= [*d_ij_*] has the singular value decomposition *U*∑*U^t ^*= *D*, then *x *= *U*∑^1/2 ^is a solution for the exact MDGP defined by *l_ij _*= *u_ij _*= *d_ij _*[3]. Even in the case where the set of known distances is incomplete, i.e., when some entries of the distance matrix *D *= [*d_ij_*] is unknown, we can solve the MDGP in linear time using an iterative algorithm called **geometric buildup **[6]. First, this algorithm initializes a set  B (base) with the index of four points, whose distances between all of them are known. Then, the coordinates of the points in  B are set using the singular value decomposition of the incomplete distance matrix *D *restricted to the base  B, and the remaining unset coordinates *x_j _*are calculated by solving the linear system

(1) $$\langle x_{i},\;x_{j}\rangle =\frac{d_{i,1}^{2}-d_{i,j}^{2}+d_{j,1}^{2}}{2},$$

where $i\in \left\{i_{1},i_{2},i_{3},i_{4}\right\}\subset B$ and *d_ij _*= ||*x_j _*- *x_i_*||. The indexes *i*_1_, *i*_2_, *i*_3_, *i*_4 _can be chosen in an arbitrarily way, allowing us to choose another base subset when calculating the coordinate of the next *x_j_*. At each iteration, the index *j *of the new coordinate *x_j _*is inserted in the set  B increasing the number of subsets {*i*_1_, *i*_2_, *i*_3_, *i*_4_} used as anchors to fix the remaining unset coordinates.

Unfortunately, in practice, the NMR experiments just provide a subset of distances between atoms spatially close and the data accuracy is limited. Thus in the real scenario, the set *E *is sparse and *l_ij _< u_ij_*. So, we just have bounds to some of the entries of the distance matrix *D*. In this situation, neither the singular value decomposition nor the **buildup **algorithm can be applied directly because they are both designed to deal with exact distances. In fact, the inaccurate and sparse instances of MDGP, where *l_ij _*<*u_ij_*, are much harder to solve as pointed by Moré and Wu who showed that the MDGP with inaccurate distances belongs to the NP-hard class of problems [7].

Our contribution is a new algorithm that can handle with inaccurate and sparse distance data. We propose an iterative method based on simple ideas: generate an approximated distance matrix *D*, take as base a clique in the graph that has *D *as a connectivity matrix, solve the system (1) and refine the solution using a nonlinear least-squares method. It needs to be pointed that the authors of the **buildup **algorithm and coworkers have done some modifications in the original form of the algorithm in order to handle inaccurate data [8,9]. However, the main advantage of our proposal is its simplicity and robustness. We have been able to find solutions with acceptable quality to instances of MDGP with inaccurate and sparse data, considering up to thousands of atoms.

## The new iterative method

### Defining the initial base

The set *E *of pairs (*i*, *j*) and the set of indexes *V *= {1, 2, ..., *n*} can be considered as a set of edges and a set of vertexes of a graph *G *= (*V*, *E*), respectively. One may decide to use as base the biggest complete subgraph of *G*. The problem of calculating the biggest complete subgraph belongs to the NP-complete class and it has a large number of applications (for a review in this subject consult [10]). We decided to use the algorithm **cliquer **proposed by Östergård in [11,12] mainly because its good behavior in graphs of moderately size and its availability on the Internet [13,14]. The **cliquer **algorithm uses a branch-and-bound algorithm developed by Östergård [15], which is based on an algorithm proposed by Carraghan and Pardalos [16].

### Setting the coordinates

Once we have obtained the base  B associated with a complete subgraph using the algorithm **cliquer**, we need to set its coordinates. In order to generate an approximated Euclidean distance matrix (EDM) restricted to the points in the base, we define a matrix *D*(*t*) = [*d_ij_*(*t*)], where

(2) $$d_{ij}\left(t\right)=\left(1-t_{ij}\right)l_{ij}+t_{ij}u_{ij}$$

for *t_ij _*∈ [0, 1] for each (*i*, *j*) ∈ *E*. With this choice, we have *l_ij _*≤ *d_ij _*≤ *u_ij_*, but *D *may not be an EDM with appropriated embedding dimension (*k *= 3). This may happen because the entries *d_ij _*can violate the triangular inequality *d_ij _*≤ *d_ik _*+ *d_jk _*for some indexes *i*, *j*, *k*, or because the rank of *D *is greater than 3. With this in mind, instead of considering the solution given by singular value decomposition directly, we take the columns (eigenvectors) of *U *associated with the 3 largest eigenvalues, getting the best 3-approximation rank of the solution to *xx^t ^*= *D*(*t*) [17].

### Refinement process

**Figure 1:**
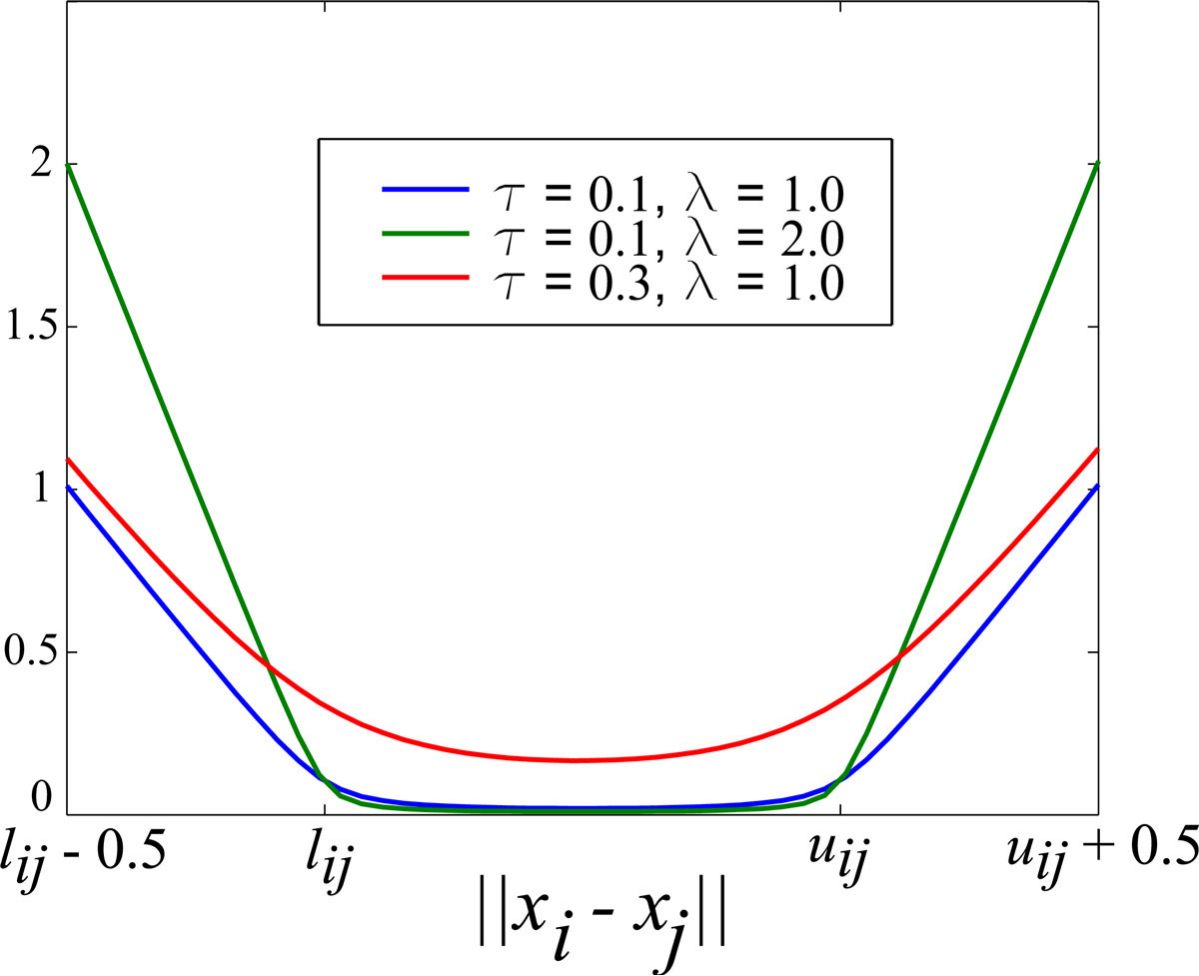
**The hyperbolic smooth penalty function**. The parameter *τ *controls the smoothness and the parameter *λ *is related to the intensity of the penalty.

We should not expect great precision in *x*, because the matrix *D*(*t*) is just an approximation. Then, we try to refine it by minimizing the nonlinear function

(3) $$\underset{x}{\text{min}}\phi_{\lambda ,\tau }\left(x\right)=\underset{\left(i,j\right)\in E:i,j\in B}{\sum }\phi_{\tau ,\lambda }^{i,j}\left(x,l,u\right),$$

where

$$\phi_{\tau ,\lambda }^{i,j}\left(x,l,u\right)=\lambda \left(l_{ij}-u_{ij}\right)+\theta_{\tau ,\lambda }^{ij}\left(x,l\right)+\theta_{\tau ,\lambda }^{ij}\left(x,u\right),$$

and

$$\theta_{\tau ,\lambda }^{ij}\left(x,c\right)=\sqrt{\lambda^{2}{\left(c-\sqrt{||x_{i}-x_{j}|{|}^{2}+\tau^{2}}\right)}^{2}+\tau^{2}},$$

with λ *>*0, *τ >*0. The parameter *τ *controls the smoothness degree and λ controls the intensity (weight) of the penalty function *φ_λ,τ _*(see Figure 1).

The function *φ_τ,λ _*is infinitely differentiable with respect to *x*, and therefore allows the application of classical optimization methods. The function *φ_τ,λ _*is a variation of the hyperbolic penalty technique used in [18,19]. In order to minimize the function *φ_τ,λ_*, we used the local minimization routine **va35 **encoded in FORTRAN and available at Harwell Subroutine Library. The routine **va35 **implements the method BFGS with limited memory [20] (For additional information on this routine, see [21]).

Once we have refined the coordinates of the points in the base  B, we start to set the remaining (free) points. We begin with the points that have at least four constraints with the points in the base. In order to set the coordinate *x_j_*, instead of using just four constraints involving the index *j *(like in the original version of the **buildup **algorithm), we use all constraints involving the index *j *and the indexes in the base. Explicitly, to set the coordinate *x_j_*, we use the approximated distance matrix *D*(*t*) for some *t *∈ [0, 1]^|*E*|^, solve the linear system

(4) $$\langle x_{i},x_{j}\rangle =\frac{d_{i,1}^{2}-d_{i,j}^{2}+d_{j,1}^{2}}{2},i\in B,$$

and then we refine the solution by minimizing the function *φ_λ,τ_*(*x*) restricted to the index *j *and to the indexes in the base (see eq. (3)). Each newly calculated coordinate is included in the base. In the end, some points may not be fixed because they have less than four constraints involving the points in the base. In this case, we just position these points solving an undetermined system defined by constraints with points in the base. Our presented ideas are compiled in the algorithm **lsbuild **(see Additional file 1).

## Methods

We have implemented our algorithm **lsbuild **in Matlab and tested it with a set of model problems on an Intel Core 2 Quad CPU Q9550 2.83 GHz, 4GB of RAM and Linux OS-32 bits. In all experiments the parameters of the function *φ_λ,τ _*of the algorithm **lsbuild **were set at *λ *= 1.0 and at *τ *= 0.01.

We compared our results with the algorithms **dgsol **and **buildup**. The algorithm **dgsol **proposed by Moré and Wu in [22] uses a continuation approach based on the Gaussian transformation

$${\langle f\rangle }_{\lambda }\left(x\right)=\frac{1}{\pi^{n/2}\lambda^{n}}\int_{{\mathbb{R}}^{n}}f\left(y\right)\text{exp}\left(-\frac{||y-x|{|}^{2}}{\lambda^{2}}\right)dy$$

of the nonsmooth function

$$f\left(x\right)=\underset{\left(i,j\right)\in E}{\sum }p_{ij}\left(x_{i}-x_{j}\right),$$

where the potentials *p_ij _*are given by

$$p_{ij}\left(x\right)=\text{max}{\left\{\frac{l_{ij}^{2}-{||x||}^{2}}{l_{ij}^{2}},\frac{{||x||}^{2}-u_{ij}^{2}}{u_{ij}^{2}},0\right\}}^{2}.$$

The algorithm **dgsol **starts with an approximated solution and, given a sequence of smoothing parameters λ_0 _> λ_1 _> ... > λ*_p _*= 0, it determines a minimizer *x*_*k*+1 _of 〈*f*〉_λ_. The algorithm **dgsol **uses the previous minimizer *x_k _*as the starting point for the search. In this manner a sequence of minimizers *x*_1_, ..., *x*_*p*+1 _is generated, with the *x*_*p*+1 _a minimizer of *f *and the candidate for the global minimizer. In our experiments, we used the implementation of the algorithm **dgsol **encoded in language C and downloaded from [23].

We also compared our results with the ones obtained by the version of the algorithm **buildup **proposed by Sit, Wu and Yuan in [8]. The algorithm **buildup **starts defining a base set using four points whose distances between all of them are known (a clique of four points). Then, at each iteration, a new point *x_k _*with known distances to at least four points in the base is selected. In order to avoid the accumulation of errors, instead of just positioning the new point, in the modified version of the algorithm **buildup **the entire substructure formed by the point *x_k _*and its neighbors in the base is calculated by solving the nonlinear system

$$\langle x_{i},x_{j}\rangle =\frac{d_{i,1}^{2}-d_{i,j}^{2}+d_{j,1}^{2}}{2},\forall i,j\in B$$

with variables $x_{i}=\left(x_{i}^{1},x_{i}^{2},x_{i}^{3}\right),x_{j}=\left(x_{j}^{1},x_{j}^{2},x_{j}^{3}\right)\in {\mathbb{R}}^{3}$ and *B *being the set formed by the index *k *and the indexes of all neighbors of *x_k _*in the current base set. The parameters *d_kj _*are the given distances between the node *x_k _*and its neighbors *x_j _*in the base and, for the nodes *x_j _*and *x_i _*already in the base, if the distance between them is unknown, we consider *d_ij _*= ||*x_i _*- *x_j_*||. Once the substructure is obtained, it is inserted in the original structure by an appropriated rotation and translation and the point *x_k _*is included in the base. This process is repeated until all nodes are included in the base. We have implemented the **buildup **algorithm in Matlab.

Our decision to compare the **lsbuild **with the algorithms **dgsol **and **buildup **is mainly motivated by theirs similarities with our proposal. In fact, the algorithm **dgsol **uses a smooth technique in order to avoid the local minimizers and the algorithm **buildup **solves a sequence of systems which produce partial solutions and iteratively try to construct a candidate to global solution. Our algorithm combines some variations of these two ideas. We use a hyperbolic smooth technique to insert differentiability in the problem and a divide-and-conquer approach based in sucessive solutions of overdetermined linear systems in order to construct a candidate to global solution.

In our experiments, the distance data were derived from the real structural data from the Protein Data Bank (PDB) [24]. It needs to be pointed that each of the algorithms considered has a level of randomness, the algorithm **dgsol **takes random start point and the algorithms **lsbuild **and **buildup **starts with an incomplete random matrix *D *= [*d_ij_*] where *l_ij _*≤ *d_ij _*≤ *u_ij_*. So, in order to do a fair comparison, we run each test 30 times.

We considered two set of instances. The first one was proposed by Moré and Wu in order to validate the algorithm **dgsol **[22]. This set is derived from the three-dimensional structure of the fragments made up of the first 100 an 200 atoms of the chain *A *of protein PDB:1GPV[25,26]. For each fragment, we generated a set of constraints considering only atoms in the same residue or the neighboring residues. Formally,

$$E=\left\{\left(i,j\right):x_{i}\in R\left(k\right),x_{j}\in \left(R\left(k\right)\cup R\left(k+1\right)\right),\forall k\right\},$$

where *R*(*k*) represents the *k*-th residue.

In this set of instances, the bounds *l_ij _*and *u_ij _*were given by the equations

$$l_{ij}=\left(1-\varepsilon \right){\hat{d}}_{ij},\;u_{ij}=\left(1+\varepsilon \right){\hat{d}}_{ij},$$

where ${\hat{d}}_{ij}$ is the real distance between the nodes *x_i _*and *x_j _*in the known structure x* of protein PDB:1GPV. In this way, all distances between atoms in the same residue or neighboring residues were considered. We generated two instances for each fragment by taking *ε *equals to 0.00 and 0.08.

In order to measure the precision of the solutions just with respect to the constraints, without providing any information about the original structure x*, we use the function

(5) $$LDME={\left(\frac{1}{\left|E\right|}\underset{\left(i,j\right)\in E}{\sum }e_{ij}^{2}\right)}^{1/2},$$

where

$$e_{ij}=\text{max}\left\{l_{ij}-||x_{i},-,x_{j}||,\;||x_{i},-,x_{j}||-u_{ij},0\right\}$$

is the error associated to the constraint *l_ij _*≤ ||*x_i _*- *x_j_*|| ≤ *u_ij_: *We also measured the deviation

of the solutions generated by each algorithm with respect to the original solution x* in the PDB files, using the function

(6) $$RMSD=\frac{1}{\sqrt{n}}\underset{Q,h}{\min}||x^{*},-,Q,(,x,-,h,)||{}_{F},$$

with $h\in {\mathbb{R}}^{n\times 3}$ and $Q\in {\mathbb{R}}^{3\times 3}$ orthogonal.

In the second experiment, we use a more realistic set of instances with larger proteins proposed by Biswas in [17]. Typically, just distances below 6Å (1Å = 10^-8 ^cm) between some pair of atoms can be measured by NMR techniques. So, in order to produce more realistic data, we considered only 70% of the distances lower than *R *= 6 Å. To introduce noise in the model, we set the bounds using the equations

(7) $$l_{ij}=d_{ij}^{*}\text{max}\left(0,1-\left|{\bar{\varepsilon }}_{ij}\right|\right),\;u_{ij}=\overset{*}{\underset{ij}{d}}\left(1+\left|{\underset{-}{\varepsilon }}_{ij}\right|\right),$$

where $d_{ij}^{*}$ is the true distance between atom *i *and atom *j *and ${\bar{\varepsilon }}_{ij},{\underset{-}{\varepsilon }}_{ij}\sim N\left(0,\sigma_{ij}^{2}\right)$ (normal distribution). With this model, we generate a sparse set of constraints and introduce a noise in the distances that are not so simple as the one used in the instances proposed by Moré and Wu.

## Results and discussion

In Table 1 we can see the results of the first experiment defined from the protein PDB:1GPV and all distances in the same or neighboring residues. The values show that the algorithms **buildup **and **lsbuild **worked better (lower *LDME *and *RMSD *and CPU time) than the algorithm **dgsol **in all instances. The algorithms **buildup **performed slightly better than the algorithm **lsbuild **being the fastest algorithm. Despite its simplicity, this set of instances worked as an indication of the correctness of our implementation of the **buildup **algorithm.

**Table 1:** RMSD, LDME and the CPU time in seconds for PDB:1GPV protein.

	Fragment with 100 atoms					
	
	*ε *= 0.00			*ε *= 0.08		
	
	〈*LDME*〉	〈*RMSD*〉	〈*TIME*〉	〈*LDME*〉	〈*RMSD*〉	〈*TIME*〉
**dgsol**	8.29E-03	3.93E-01	3.61E+00	3.31E-03	8.25E-01	4.40E+00
**buildup**	3.50E-15	1.46E-14	1.08E-01	0.00E+00	3.13E-01	1.08E-01
**lsbuild**	6.47E-15	1.20E-14	1.51E-01	0.00E+00	7.77E-02	1.33E-01

	Fragment with 200 atoms					
	
	*ε *= 0.00			*ε *= 0.08		
	
	〈*LDME*〉	〈*RMSD*〉	〈*TIME*〉	〈*LDME*〉	〈*RMSD*〉	〈*TIME*〉

**dgsol**	3.18E-02	2.58E+00	1.48E+01	4.00E-03	2.45E+00	1.73E+01
**buildup**	4.85E-15	2.45E-14	3.11E-01	0.00E+00	5.18E-01	3.11E-01
**lsbuild**	1.90E-14	5.21E-14	6.01E-01	0.00E+00	4.21E-01	5.25E-01

Results for the fragments made up with the first 100 and 200 atoms of protein PDB:1GPV. The 〈*LDME*〉 and 〈*RMSD*〉 represent the *LDME *and *RMSD *measures respectively and 〈*TIME*〉 represents the mean time in seconds.

Table 2 shows the results of the second experiment with more realistic data. We can see that our approach was more efficient than the algorithms **buildup **and **dgsol **that were not able to find good solutions in these harder instances. In this table, |*V*| is the number of atoms in the instance, and CPU time is given in seconds. We also point out that *LDME *was low and the *RMSD *was lower than 3.5Å in all instances, which means that the algorithm is robust and able to find protein structures very similar to the original ones [1]. The results in Table 3 shows that the **buildup **algorithm was again the fastest. The CPU time of the algorithm **lsbuild **was in the average around to 2.45 times the time consumed by the algorithm **buildup**, this fact must be mitigated by the better quality of the solutions obtained be the algorithm **lsbuild**.

**Table 2:** RMSD and LDME for the larger instance set.

		〈*LDME*〉			〈*RMSD*〉		
		
PDB	|*V*|	lsbuild	buildup	dgsol	lsbuild	buildup	dgsol
1PTQ	402	2.61E-03	1.80E+00	5.41E-01	1.31E-02	9.49E+00	6.89E+00
1LFB	641	2.03E-04	1.84E+00	3.91E-01	4.19E-03	1.23E+01	5.48E+00
1AX8	1003	2.00E-04	1.83E+00	4.33E-01	1.62E-02	1.35E+01	7.95E+00
1F39	1534	3.03E-02	1.89E+00	4.74E-01	4.22E-01	1.79E+01	1.28E+01
1RGS	2015	1.08E-01	1.87E+00	4.73E-01	1.74E+00	1.92E+01	1.35E+01
1KDH	2846	1.39E-02	1.86E+00	5.19E-01	9.43E-02	2.11E+01	1.61E+01
1BPM	3671	2.20E-02	1.90E+00	5.14E-01	7.86E-02	2.29E+01	1.55E+01
1TOA	4292	6.90E-03	1.89E+00	6.75E-01	2.56E-01	2.52E+01	2.39E+01
1MQQ	5681	1.93E-02	1.91E+00	8.86E-01	1.89E-01	2.50E+01	2.50E+01

Results with instances considering just 70% of the distances below 6Å. The 〈*LDME*〉 and 〈*RMSD*〉 represent the mean *LDME *and mean *RMSD *respectively.

**Table 3:** TIME for the larger instance set.

	〈*TIME*〉		
	
PDB	lsbuild	buildup	dgsol
1PTQ	9.99E-01	5.34E-01	1.03E+01
1LFB	1.86E+00	1.01E+00	2.55E+01
1AX8	2.98E+00	1.70E+00	4.36E+01
1F39	7.21E+00	3.57E+00	8.59E+01
1RGS	1.43E+01	4.70E+00	1.33E+02
1KDH	2.12E+01	7.28E+00	2.09E+02
1BPM	2.47E+01	8.04E+00	2.99E+02
1TOA	3.93E+01	1.14E+01	7.03E+02
1MQQ	3.93E+01	1.82E+01	7.63E+02

The mean CPU time in seconds with the instances considering just 70% of the distances below 6Å.

Finally, the results of both set of instances indicate that our algorithm **lsbuild **based on the combination of the resolution of linear systems, derived from the approximated EDM matrices, and the refinement process based on hyperbolic smoothing penalty is a very effective strategy to solve MDGP instances with sparse and inaccurate data.

## Conclusions

We presented a new algorithm to solve molecular distance geometry problems with inaccurate distance data. These problems are related to molecular structure calculations using data provided by NMR experiments which, in fact, are not precise. Our algorithm combines the divide-and-conquer framework and a variation of the hyperbolic smoothing technique. The computational results show that the proposed algorithm is an effective strategy to handle uncertainty in the data.

## Competing interests

The authors declare that they have no competing interests.

## Authors' contributions

MS, AM and CL participated in the development of the ideas presented in the design of the proposed algorithm. MS and CL drafted the manuscript. CL and NM gave final approval of the version to be published. All authors read and approved the final manuscript.

## Supplementary Material

Additional file 1 **Algorithm lsbuild**. Click here for file

## Declarations

The publication of this article is supported by the Brazilian Research Agencies FAPESP and CNPq.
