# Supplementary material for: Solving the molecular distance geometry problem with inaccurate distance data
Source: BMC Bioinformatics. 2013 Jun 28;14(Suppl 9):S7. doi: 10.1186/1471-2105-14-S9-S7 (PMC3698034; doi:10.1186/1471-2105-14-S9-S7)
Supplement: Additional file 1 — Algorithm lsbuild. [file 1471-2105-14-S9-S7-S1.pdf]

Algorithm 1 An iterative algorithm to solve the MDGP with inaccurate data

procedure lsbuild(V, E, l, u)
  $D = [d_{ij}] = [t_{ij} l_{ij} + (1 - t_{ij}) u_{ij}], \forall (i, j) \in E$; ▷ Approximated EDM with $t_{ij} \in [0, 1]$
  $\mathcal{B} = \textbf{cliquer}(G(V, E))$; ▷ Initial base (clique).
  $U \Sigma U^t = svd(D')$, where $D' = [d_{ij}], \forall (i, j) \in \mathcal{B}$;
  Define $\tilde{\Sigma}$ as the diagonal matrix with the three biggest eigenvalues of Σ;
  Define $\tilde{U}$ as the matrix with the columns of U associated to the eigenvalues in $\tilde{\Sigma}$;
  $x_j = [\tilde{U} \Sigma^{1/2}]_j, j \in \mathcal{B}$; ▷ $[M]_j$ represents the j-th row of the matrix M.
  Refine $x = [x_j], j \in \mathcal{B}$ using the model given by the eq. (3);
  $L = V - \mathcal{B}$; ▷ List of non fixed coordinates.
  while L is not empty **do**
    $K = \{j \in L :$ node j has at least four neighbors in $\mathcal{B}\}$;
    for $j \in K$ **do**
      Solve the linear system given by the eq. (4);
    end for
    $L = L - K$; ▷ Update L
    $\mathcal{B} = \mathcal{B} \cup K$; ▷ Update $\mathcal{B}$
    Refine $x = [x_j], j \in \mathcal{B}$ using the model given by the eq. (3);
  end while
end procedure
